# Cepharanthine Inhibits *Fusarium solani* via Oxidative Stress and CFEM Domain-Containing Protein Targeting

**DOI:** 10.3390/microorganisms13061423

**Published:** 2025-06-18

**Authors:** Yuqing Wang, Zenghui Yang, Jingwen Xue, Yitong Wang, Haibo Li, Zhihong Wu, Yizhou Gao

**Affiliations:** School of Biological and Chemical Engineering, Zhejiang University of Science and Technology, Zhejiang Provincial Collaborative Innovation Center of Agricultural Biological Resources Biochemical Manufacturing, Hangzhou 310023, China

**Keywords:** cepharanthine, *Fusarium solani*, antifungal activity, machine learning, CFEM domain-containing protein, natural compound

## Abstract

Cepharanthine (CEP) is a natural bisbenzylisoquinoline alkaloid known for its antibacterial, antiviral, and anti-inflammatory activities. Its antifungal effect, however, has not been well studied. In this work, we used machine learning-based virtual screening with Random Forest, Neural Network, and Support Vector Machine models to identify potential inhibitors of *Fusarium solani*. CEP was selected as a candidate and tested experimentally. The results showed that it inhibited the growth of *Fusarium solani*, *Fusarium proliferatum*, *Fusarium oxysporum*, *Alternaria alternata*, and *Botrytis cinerea*. It also reduced the sporulation and spore germination of *Fusarium solani* and disrupted its redox balance. Transcriptome analysis showed changes in gene expression related to basic metabolic pathways. Molecular docking suggested that CEP binds to the FsCFEM1 protein, and molecular dynamics simulations confirmed stable binding, with key roles for residues THR748 and LEU950. These results suggest that CEP is a potential bio-based antifungal agent and provide novel insights into its mechanism against *Fusarium solani*.

## 1. Introduction

*Fusarium solani* is a widespread soilborne pathogen that causes root rot and wilt in many crops, including alfalfa, tobacco, sweet potato, and peanut [1]. These diseases impair plant growth, reduce yields, and affect product quality. At present, chemical fungicides such as carbendazim and procymidone are the main means of control [2]. However, the long-term and extensive use of these fungicides has led to environmental pollution, the development of resistant strains, and food safety concerns, which conflict with the goals of sustainable agriculture [3]. Biological control strategies have drawn increasing attention as alternatives to chemical fungicides. Several endophytic fungi, such as *Trichoderma reesei* and *Chaetomium globosum*, have shown the ability to inhibit the mycelial growth of *F. solani* in vitro [4,5]. In addition, plant-derived secondary metabolites, including carvacrol and eugenol, exhibit antifungal activity by disrupting cell wall and membrane structures, altering their morphology, and interfering with ergosterol biosynthesis [6,7]. Seaweed extracts and certain biofertilizers have also shown inhibitory effects on *F. solani*, with reduced spore germination observed at higher concentrations [8,9]. Despite these findings, the field application of antagonistic microorganisms and natural compounds remains limited. There is an urgent need to develop efficient, safe, and broad-spectrum agents for managing *F. solani*.

Natural products and microbial metabolites are important sources for discovering new antifungal compounds [10]. In recent years, machine learning has been applied in virtual screening, allowing the analysis of large chemical libraries based on structural and biological data to predict antifungal activity [11,12,13]. Unlike traditional experimental screening, which is often time-consuming and resource-intensive, machine learning-based virtual screening allows the rapid, large-scale identification of potential compounds with improved efficiency and accuracy. Models trained on peptide datasets have been used to extract key activity-related features, with the Random Forest algorithm showing a relatively high performance in prediction tasks [14]. Structural biology and molecular modeling approaches, including docking and dynamics simulations, have also been used to identify candidate compounds against plant pathogens such as cotton pests and *Rhizoctonia solani* [15,16]. Furthermore, machine learning can assist in the interpretation of fungal genomic and transcriptomic data to uncover gene networks affected by antifungal agents, supporting the identification of potential targets [17]. Together, these tools provide a valuable platform for screening and characterizing antifungal candidates against *F. solani*.

Cepharanthine (CEP) is a bisbenzylisoquinoline alkaloid extracted from the roots of *Stephania* species (*Menispermaceae*). It has demonstrated broad-spectrum antibacterial, antiviral, and anti-inflammatory activities [18,19]. CEP can activate type I interferon signaling to enhance host antiviral responses and has shown inhibitory effects on viruses such as human immunodeficiency virus (HIV), hepatitis B virus (HBV), influenza A virus (H1N1), and herpes simplex virus type 1 (HSV-1) [20,21,22,23]. It also modulates the MAPK and NF-κB pathways, suppresses inflammatory cytokine expression, restores autophagy, and contributes to cellular protection [24,25]. Additionally, CEP has been reported to inhibit tumor cell proliferation, induce apoptosis and autophagy, and prevent viral entry [26]. Although its pharmacological effects are well-documented, its antifungal potential, especially against plant pathogenic fungi, remains unclear.

In this study, we constructed virtual screening models using Random Forest (RF), Neural Network (NN), and Support Vector Machine (SVM) algorithms to identify potential inhibitors of *F. solani.* These models were selected to leverage their complementary strengths, as they represent distinct classes of algorithms (ensemble, neural-inspired, and kernel-based, respectively) commonly used in biological prediction tasks [27,28]. CEP was identified as a promising candidate and subsequently validated through in vitro assays. The results showed that CEP significantly inhibited the mycelial growth of *F. solani*, *Fusarium proliferatum*, *Fusarium oxysporum*, *Alternaria alternata*, and *Botrytis cinerea*. We further investigated its effects on the sporulation, spore germination, and oxidative stress response in *F. solani*. Transcriptome analysis revealed significant changes in gene expression following CEP treatment. Molecular docking indicated a potential interaction between CEP and the FsCFEM protein, which was further supported by molecular dynamics simulations. Key residues THR748 and LEU950 were found to contribute to the stability of the binding.

## 2. Materials and Methods

### 2.1. Data Preprocessing

The relevant information of *Fusarium solani* was collected from the CHEMBL database (https://www.ebi.ac.uk/chembl/) (accessed on 28 July 2023) as the dataset for the construction of the machine learning model, and 1275 relevant active compounds’ molecular information was obtained. The modeling dataset was cleaned and proofread, and the values of duplicate molecule ChEMBL IDs were deleted, the unified minimum inhibitory concentration (MIC) unit was calculated, and the compounds with no MIC or multiple MICs were deleted. Generally, when essential oils, alkaloids, and other substances are used for the biocontrol of certain pathogenic fungi, they are considered to have inhibitory effects if they can suppress the fungi at concentrations lower than 50 µg/mL [29,30,31]. Isoeugenol exhibited considerable efficacy against free radicals, with MIC50 values of 38.97 and 43.76 µg/mL [30]. Inhibitors and non-inhibitors were divided according to their MIC value, and compounds with a MIC < 50 µg/mL were marked as 0 (representing inhibitors), and those with a MIC ≥ 50 µg/mL were marked as 1 (representing non-inhibitors).

### 2.2. Molecular Characterization, Feature Selection, and Dataset Partitioning

Descriptors and MoleculeDescriptors of the toolkit RDKit in Python 3.7.0 were used to batch-calculate descriptors based on the Simplified Molecular Input Line Entry System (SMILES) of the chemical structure of the compounds in the modeling dataset, and all descriptors were obtained using Descriptors._descList, a component package within Python 3.7.0. Feature dimension reduction was performed by Recursive Feature Elimination (RFE), and 50 molecular descriptors were finally retained for the construction of the machine learning model. A tree-based ensemble model was selected as the basic model for RFE to evaluate features’ importance, and the feature set and target variable were initialized; the final number of features and the number of features eliminated in each iteration were set to 50 and 1, respectively; then all the features and target variables were used to build and train the model, and the importance scores (feature_importances_) of each feature were calculated based on the model. Several features with the lowest scores were removed to obtain a new feature set, and the steps of construction of the model, evaluation of importance, and feature elimination were repeated until the predetermined number of features was reached, the model performance was no longer improved, or the difference in feature importance was less than the threshold, and the feature set that was finally left was the selected optimal feature subset. The dataset was normalized using the Standard scalar function in the Python 3.9 toolkit Scikit-learn and then divided into a training set and a test set in a 4:1 ratio.

### 2.3. Grid Search and Five-Fold Cross-Validation

In order to achieve the best performance of the model, the grid search algorithm is used to determine the optimal parameter combination of RF, SVM, and NN. The three models set the hyperparameter space, clarify the hyperparameters to be optimized, and specify a set of possible value ranges for each hyperparameter, respectively. For RF, n_estimators, max_depth and min_samples_leaf, were selected as the hyperparameters to be optimized; for SVM, the hyperparameters to be optimized were gamma and C; for the NN model, hidden_layer_sizes and max_iter were selected as the hyperparameters to be optimized. The training set was randomly divided into five non-overlapping subsets, each containing approximately 20% of the total data volume. These subsets maintained data distribution consistency as much as possible to ensure that each fold contained inhibitor and non-inhibitor data; each time, one of the subsets was selected as the test set, and the remaining four subsets were combined as the training set. Each subset had the opportunity to be used as a test set once and also as a training set four times; in each round, the performance indicators of the model on the test set were recorded; finally, after five training and validation rounds, the average values of the performance indicators were calculated, including accuracy, precision, recall, F1 score, and Area Under the receiver operating characteristic Curve (AUC), and the performance of each model was carefully compared.

### 2.4. Assessment of Mycelial Growth, Conidiation, and Spore Germination

Following the methodology described previously [32], *Fusarium solani*, *Fusarium oxysporum*, *Fusarium proliferatum*, *Botrytis cinerea*, and *Alternaria alternata* were cultured and treated with CEP (Macklin, Shanghai, China). Each fungal strain was inoculated onto PDA medium supplemented with different concentrations of CEP (10 mg/L, 20 mg/L, 30 mg/L, 40 mg/L, 50 mg/L, 60 mg/L, 80 mg/L, 100 mg/L, 120 mg/L, 200 mg/L, 250 mg/L, 300 mg/L), while potato dextrose agar (PDA) medium without CEP was used as a control. The cultures were incubated at 26 °C for 7 days, and colony diameters were measured to calculate the mycelial growth inhibition rate. For conidiation assays in *F. solani*, the fungus was inoculated into mung bean broth supplemented with 200 mg/L CEP and incubated at 26 °C with shaking at 250 rpm for 2 days. The number of conidia produced was determined using a hemocytometer. To assess conidial germination, conidia were harvested from 2-day-old *F. solani* cultures and suspended in YEPD medium (3 g yeast extract, 10 g peptone, and 20 g glucose per liter) supplemented with 200 mg/L CEP. The cultures were incubated at 26 °C with shaking for 6 and 12 h, respectively. At each time point, at least 100 randomly selected conidia per field of view were examined under a microscope to determine their germination rates. Statistical significance analysis was performed using a one-way analysis of variance (ANOVA) with pairwise comparisons, implemented via the SPSS 21.0 statistical software package.

### 2.5. Mycelial Preparation and Oxidative Stress Assays

To evaluate the oxidative stress of *F. solani* after treatment with 20 mg/L, 50 mg/L, 100 mg/L, and 200 mg/L CEP, malondialdehyde (MDA) content, hydrogen peroxide (H_2_O_2_) content, and the activities of superoxide dismutase (SOD), peroxidase (POD), and catalase (CAT) were measured. First, an *F. solani* spore suspension was prepared and adjusted to 1 × 10^6^ spores/mL. A 1 mL aliquot was inoculated into 100 mL of potato dextrose broth (PDB) and incubated at 26 °C with shaking at 200 rpm for 2 days. The mycelia were collected by filtration, washed with sterile water, and transferred into culture media containing 20 mg/L, 50 mg/L, 100 mg/L, or 200 mg/L CEP. Untreated mycelia served as controls. After incubation for 12 h with shaking, the mycelia were collected by vacuum filtration, washed with sterile water, and used for subsequent assays. For MDA content’s measurement, the thiobarbituric acid (TBA) (Sigma-Aldrich, St. Louis, MO, USA) method was used. A 1.0 g mycelial sample was ground into powder in liquid nitrogen, followed by the addition of 5.0 mL of 10% trichloroacetic acid (TCA) (Sigma-Aldrich, St. Louis, MO, USA). After homogenization, the mixture was centrifuged at 10,000× *g* for 20 min at 4 °C, and the supernatant was collected. A 2.0 mL aliquot of the supernatant (for the blank control, 2.0 mL of 10% TCA was used instead) was mixed with 2.0 mL of 0.67% TBA solution, heated in a boiling water bath for 20 min, cooled, and centrifuged again. The absorbance of the supernatant was measured at 450 nm, 532 nm, and 600 nm. For the H_2_O_2_ content’s measurement, a commercial hydrogen peroxide assay kit (Biyuntian, Shanghai, China) was used, following the manufacturer’s instructions. For antioxidant enzyme activity assays, SOD activity was measured using a SOD assay kit (Solarbio, Beijing, China). For the POD activity assay, a 1 g mycelial sample was ground into powder in liquid nitrogen, suspended in 1 mL of PBS (pH 7.2) (Sigma-Aldrich, St. Louis, MO, USA), and centrifuged at 4000 rpm for 10 min. The supernatant was collected, and POD activity was determined using a POD assay kit (Solarbio, Beijing, China). For the CAT activity assay, a CAT assay kit (Solarbio, Beijing, China) was used.

### 2.6. Chitin Content Measurement

To investigate the effect of CEP on fungal cell wall integrity, the chitin content in *F. solani* mycelia was determined. A 5 mg sample of ground mycelial powder was suspended in 1 mL of 6% KOH solution (Aladdin, Shanghai, China). The sample was incubated in an 80 °C water bath for 1.5 h, followed by centrifugation at 12,000 rpm for 10 min. The supernatant was discarded, and the pellet was resuspended in 1 mL of 10 mM phosphate-buffered saline (pH 7.4). The washing step was repeated twice under the same centrifugation conditions. The final pellet was resuspended in 100 μL of McIlvaine buffer (pH 6.0) (Sigma-Aldrich, St. Louis, MO, USA) with 5 μL of chitinase and incubated at 37 °C for 24 h. Following enzymatic hydrolysis, 100 μL of 0.27 M boric acid solution (Sigma-Aldrich, St. Louis, MO, USA) was added, and the sample was boiled for 10 min before cooling it to room temperature. After adding 1 mL of dimethylaminobenzaldehyde (DMAB) (Aladdin, Shanghai, China) solution, the mixture was incubated at 37 °C for 20 min, and absorbance was measured at 585 nm. A standard curve was generated using N-acetylglucosamine (0.05–0.40 mM) (Sigma-Aldrich, St. Louis, MO, USA) to determine the chitin content.

### 2.7. RNA Extraction

For RNA extraction, at least 0.5 g of *F. solani* mycelia from CEP-treated samples was collected, along with untreated mycelia as controls. Each group included three biological replicates. The collected mycelia were ground into fine powder in liquid nitrogen and transferred into 2 mL centrifuge tubes. A total of 1 mL of TRIzol reagent (Vazyme Biotech, Nanjing, China) was added to each tube, followed by thorough mixing and incubation at room temperature for 10 min. Then, 200 μL of chloroform (Sigma-Aldrich, St. Louis, MO, USA) was added, and the mixture was shaken at 70 Hz for 120 s, followed by another 10 min of incubation at room temperature. The sample was centrifuged at 12,000 rpm for 10 min at 4 °C, and the aqueous phase was transferred to a new tube. An additional 200 μL of chloroform was added for a second extraction, followed by the same centrifugation step. The final aqueous phase was mixed with an equal volume of isopropanol (Sigma-Aldrich, St. Louis, MO, USA) and incubated on ice for 1 h. The RNA was then precipitated by centrifugation at 12,000 rpm for 10 min at 4 °C. The supernatant was discarded, and the RNA pellet was washed with 1 mL of 70% ethanol prepared with DEPC-treated water (Sigma-Aldrich, St. Louis, MO, USA), followed by centrifugation at 12,000 rpm for 5 min at 4 °C. After discarding the supernatant, the pellet was air-dried in a biosafety cabinet and dissolved in 70 μL of DEPC-treated water. The RNA samples were either stored at −80 °C or used immediately for further experiments. RNA quality was assessed by agarose gel electrophoresis.

### 2.8. Transcriptome Analysis

For the construction of the RNA sequencing (RNA-seq) library, the NEB or strand-specific method was used, ensuring a library concentration above 2 nM. The insert size of the library was also evaluated before the sequencing. The sequencing reads were aligned to the *F. solani* reference genome using HISAT2 with paired-end clean reads. Gene expression levels were quantified using StringTie v2.0.4, employing both fragments per kilobase per million reads (FPKM) and transcripts per million (TPM) as expression metrics. After gene quantification, expression values from all the samples were merged into an expression matrix. Differential expression analysis was performed using DESeq2 v1.26.0, with significance criteria set as a *p*-value ≤ 0.05 and |log_2_FC| ≥ 1 for DEGs between CEP-treated and control groups. GO enrichment analysis of DEGs was conducted using the clusterProfiler package v3.14.0, with significantly enriched GO terms identified based on a *p*-value threshold of ≤0.05. KEGG pathway enrichment analysis was performed using hypergeometric testing, with pathways considered significantly enriched at a *p*-value ≤ 0.05. For functional annotation using the Clusters of Orthologous COG database, the protein sequences of DEGs were aligned against the COG database using BLASTP v2.2.31 with an E-value threshold of ≤10^−5^. Homologous COG protein clusters were identified, and the functional classification of DEGs was performed accordingly.

### 2.9. Molecular Docking and Molecular Dynamics Simulation

The structure file of the CEP small molecule was obtained from the PubChem database. The three-dimensional structure of the FsCFEM1 protein was predicted using SWISS-MODEL (https://swissmodel.expasy.org/) (accessed on 12 September 2024), and the corresponding structure file was retrieved. Molecular docking was performed using AutoDock software 4.2.6. Before docking, both the protein and the small molecule were preprocessed by removing unnecessary atoms, adding hydrogen atoms, and assigning charges. Docking parameters, including the search space, sampling algorithm, and scoring function, were set accordingly. The CFEM domain region (amino acids 725–789) of FsCFEM1 was selected as the docking box, and a semi-flexible molecular docking approach was adopted. After docking, binding modes were evaluated, and the conformations with reasonable binding poses and high docking scores were selected for further analysis. For the selected protein–ligand complexes, molecular dynamics simulations were performed using Gromacs 2022. The Amber99sb-ildn force field was applied for the protein, while the General Amber Force Field (GAFF) was used for the small molecule. A TIP3P water model was used to construct a 10 × 10 × 10 nm^3^ water box, ensuring at least 1.2 nm between the protein and the box edges. Ions were added to neutralize the system. During the simulation, long-range electrostatic interactions were treated using the particle–mesh Ewald (PME) method, and energy minimization was conducted for 50,000 steps using the steepest descent algorithm, with Coulomb and van der Waals cutoff distances set to 1 nm. After the system’s minimization, equilibration was performed under the NVT (constant number of particles, volume, and temperature) and NPT (constant number of particles, pressure, and temperature) ensembles. MD simulation was conducted for 100 ns at 300 K, controlled by the Langevin thermostat, and 1 bar, controlled by the Berendsen barostat. A 10 Å cutoff was used for non-bonded interactions. Post-simulation analysis was conducted using built-in Gromacs analysis tools. Structural stability and flexibility were assessed by calculating RMSD, RMSF, and Rg. The binding free energy was estimated using Gmx_MMPBSA, a component within Gromacs 2022.

## 3. Results

### 3.1. Construction and Evaluation of Machine Learning Models

**Figure 1:**
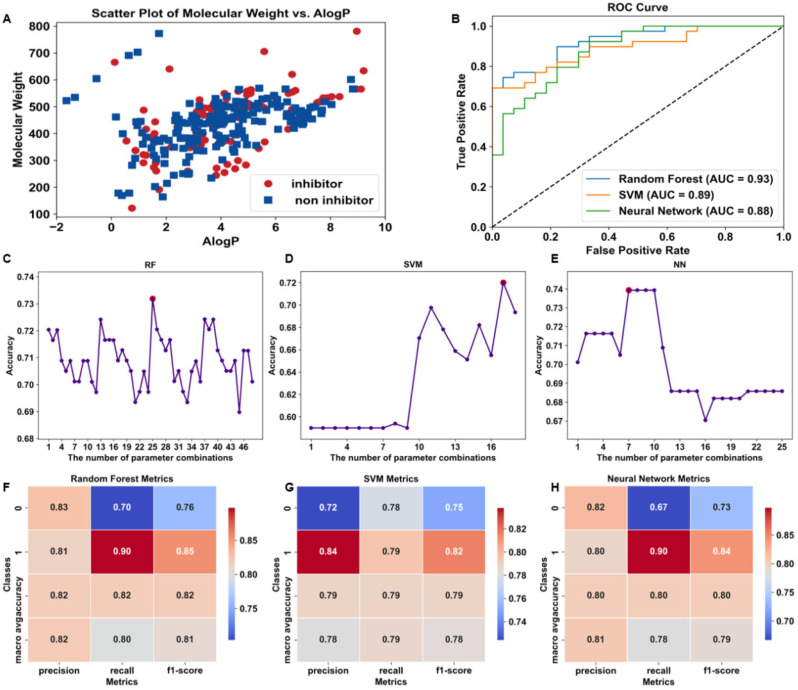
Machine learning model comparison and hyperparameter optimization in chemical space analysis. (**A**) Chemical space analysis of modeling datasets. (**B**) ROC curves of RF, SVM, and NN models. (**C**–**E**) RF, SVM, and NN find the best parameters through grid search according to the corresponding combination of hyperparameters to be optimized. RF: n_estimators = 150, max_depth = 8, min_samples_leaf = 1; SVM: gamma = 0.01, C = 100, probability = True; NN: hidden_layer_sizes = (50, 50), max_iter = 500. (**F**–**H**) Confusion matrix heat map of the mean performance index of the training set and the performance index value of the test set of the three models of RF, SVM, and NN with 5-fold cross-validation.

In this study, three machine learning algorithms, namely Random Forest (RF), Support Vector Machine (SVM), and Neural Network (NN), were selected to build models for screening compounds that inhibit *F. solani*. Before the model building, a chemical space analysis was conducted based on the Molecular Weight (MW) and Aliphatic and Aromatic LogP (AlogP) of the compounds in the processed dataset (Figure 1A). The results showed that the MW of the modeling dataset was concentrated in the range of 100 to 600, and the AlogP values ranged from 0 to 8. This indicates that the chemical space of the compounds in the modeling dataset of this study is relatively large, with a good stability and high operability, and it may contain various types of candidate drugs. In order to effectively improve the stability and reliability of the model, the 208 molecular descriptors obtained through molecular characterization calculations were screened using recursive feature elimination (RFE); the number of retained features was determined based on the RFE; when the number of features was 50, the evaluation metrics, such as the accuracy of the model on the dataset, were relatively high (Figure S1). Features at this dimension can ensure the generalizability of the models. The Pearson correlation coefficient of these 50 features was calculated (Figure S2). The results showed that the absolute values of correlation coefficients for most features were less than 0.5, indicating a weak correlation among those features. The features retained by RFE effectively removed redundancy, which served to improve the performance and accuracy of the model. The three selected machine learning models were generated using the open-source toolkit Scikit-learn in Python 3.9. The parameters of the models were adjusted using grid search, learning curves, and accuracy values to achieve the best prediction results (Figure 1C–E). Finally, the five-fold cross-validation results on the training set and the test set results of the generated machine learning models are shown in Table 1 and Figure 1F–H, respectively. The accuracy rates of the three models on the training set are close to those on the test set, indicating that the models exhibit a good generalizability and robustness. RF and SVM had similar prediction effects on inhibitors (“0”) and non-inhibitors (“1”), and NN had better prediction effects on non-inhibitors. The F1 scores and precision indicators of the three models are all greater than 0.7, indicating that the model performance is balanced and can accurately predict compounds that inhibit the activity of *F. solani* within a certain range. Additionally, this study found that the ROC curves of the three models of RF, SVM, and ANN were highly similar, and the values of the area under curve (AUC) were all above 0.80. Among them, RF had the highest AUC of 0.93, indicating that the three models performed well. Considering that different algorithms have preferences and the generalizability of a single model is insufficient, this paper takes the predictive values of the three models into comprehensive consideration for predicting whether a drug is an inhibitor for *F. solani* to improve the accuracy and reliability of the prediction.

### 3.2. Compound Screening Based on Machine Learning Models

**Figure 2:**
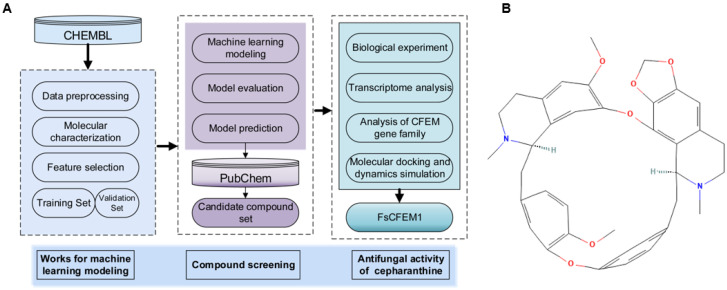
Integrated workflow and structural characterization of CEP. (**A**) Workflow of this study. (**B**) Two-dimensional chemical structure of CEP.

After generating the machine learning model and evaluating its predictive performance, 2845 natural product compounds in the PubChem database were used as the prediction dataset for the virtual screening based on the three machine learning models. The compounds in the prediction set were subjected to the same data preprocessing, molecular characterization, and feature selection (Figure 2A). The average accuracy of the three machine learning models predicting the inhibitor compounds was used for the screening. There were 248 compounds predicted as inhibitors by the three machine learning models at the same time, of which 14 compounds had prediction values greater than 0.80 [33] for all three models (Table S1). Among the 14 top-ranked candidates, CEP was selected due to its well-documented broad-spectrum antibacterial, antiviral, and anti-inflammatory activities reported in previous studies [18,19], as well as its commercial availability and low procurement cost (Figure 2B). Other candidates may be pursued in future studies.

### 3.3. Inhibitory Effects of CEP on Mycelial Growth of Plant Pathogenic Fungi

**Figure 3:**
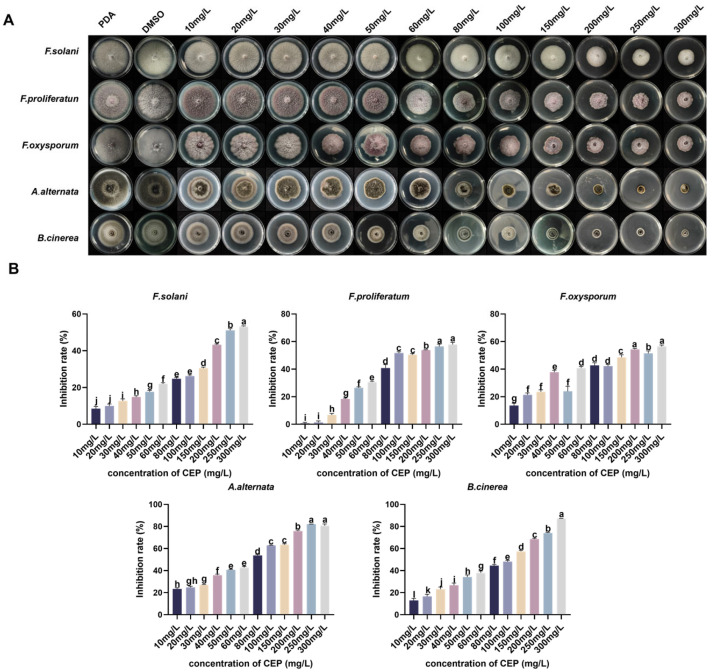
Impact of CEP on mycelial growth of plant pathogenic fungi. (**A**) Representative images of fungal colonies treated with different concentrations of CEP (0–300 mg/L). Five plant pathogenic fungi, *Fusarium solani*, *Fusarium proliferatum*, *Fusarium oxysporum*, *Alternaria alternata*, and *Botrytis cinerea*, were cultured on PDA medium supplemented with dimethyl sulfoxide (DMSO) or increasing concentrations of CEP. (**B**) Inhibition rate (%) of fungal mycelial growth in response to CEP treatment. The inhibition rate was calculated as [(control colony diameter–treated colony diameter)/control colony diameter] × 100%. Different letters indicate statistically significant differences (*p* < 0.05) according to Duncan’s multiple range test. Data are presented as the mean ± SD from three independent experiments.

To verify the antifungal activity of the selected compound CEP, a growth inhibition assay was conducted. Mycelia were treated with different concentrations of CEP (10 mg/L, 20 mg/L, 30 mg/L, 40 mg/L, 50 mg/L, 60 mg/L, 80 mg/L, 100 mg/L, 120 mg/L, 200 mg/L, 250 mg/L, and 300 mg/L), and the mycelial growth rate was observed (Figure 3A). The results showed that CEP at 10 mg/L inhibited *F. solani* by 8.5%. As the CEP concentration increased, the inhibition rate gradually increased, reaching up to 53% at 300 mg/L (Figure 3B). In addition, the sensitivity of *F. oxysporum*, *F. proliferatum*, *A. alternata*, and *B. cinerea* to CEP was also evaluated (Figure 3B). The results indicated that CEP exhibited inhibitory activity against the all tested plant pathogenic fungi, with the inhibition effect being concentration-dependent within a certain range. Among these fungi, *A. alternata* was the most sensitive to CEP, with an inhibition rate of 23.5% at 10 mg/L. These findings suggest that CEP possesses broad-spectrum antifungal activity against plant pathogenic fungi.

### 3.4. CEP Regulates Sporulation, Spore Germination, and Antioxidant Systems of Fusarium solani

**Figure 4:**
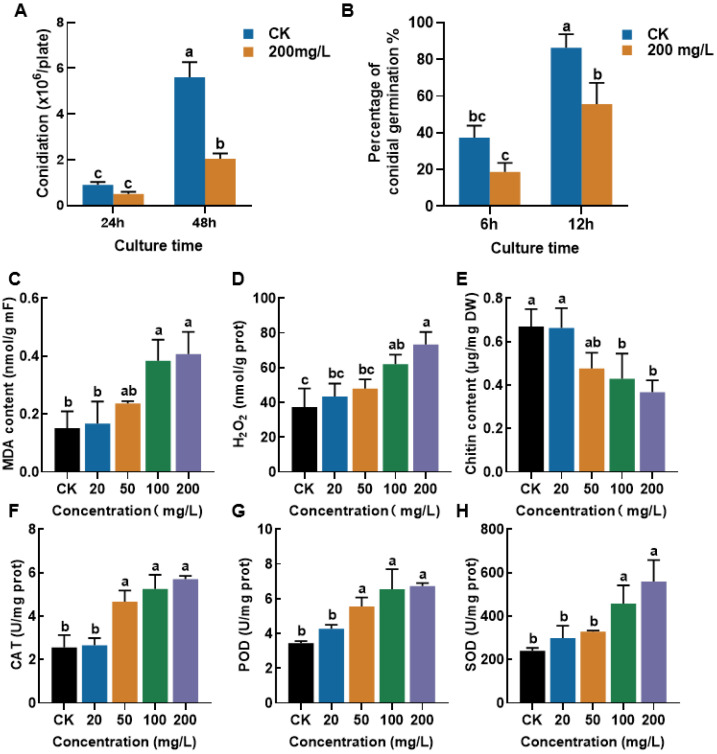
Effects of CEP on sporulation, spore germination, and oxidative stress responses in *F. solani*. (**A**) Sporulation of *F. solani* at 24 h and 48 h in the control (CK) and 200 mg/L CEP-treated groups. Data are presented as the number of conidia per plate (×10⁶). (**B**) Percentages of spore germination of *F. solani* at 6 h and 12 h in the CK and 200 mg/L CEP-treated groups. (**C**–**H**) Effects of different CEP concentrations (0, 20, 50, 100, 200 mg/L) on malondialdehyde (MDA) content C, hydrogen peroxide (H_2_O_2_) content (**D**), chitin content (**E**), catalase (CAT) activity (**F**), peroxidase (POD) activity (**G**), and superoxide dismutase (SOD) activity (**H**) in *F. solani*. Different letters above the bars indicate significant differences (*p* < 0.05) among different treatment groups.

The effects of CEP on the sporulation, spore germination, and related physiological and biochemical parameters of *F. solani* were further investigated. As shown in Figure 4A,B, compared to the control group, the sporulation of *F. solani* was significantly reduced after 48 h under treatment with 200 mg/L CEP, and the spore germination rates at 6 h and 12 h were significantly lower than those of the control group. However, no significant differences in the morphology of hyphae and spores were observed after a 48 h treatment with CEP under light microscopy (Figure S3). In the determination of several physiological and biochemical indexes, the malondialdehyde (MDA) content of the mycelia increased with the rise in CEP concentration, indicating an increase in the lipid peroxidation of the fungal cells (Figure 4C). The hydrogen peroxide (H_2_O_2_) content was significantly elevated under CEP treatment (Figure 4D). The chitin content decreased with increasing CEP concentrations, suggesting that CEP treatment may affect the composition of the fungal cell wall (Figure 4E). Moreover, treatment with CEP at concentrations above 50 mg/L significantly increased the activities of catalase (CAT) (Figure 4F) and peroxidase (POD) (Figure 4G), while the activity of superoxide dismutase (SOD) was significantly enhanced at concentrations above 100 mg/L (Figure 4H), indicating that CEP treatment influenced the antioxidant defense system of *F.solani*. In conclusion, CEP significantly affected sporulation, spore germination, and oxidative metabolic processes in *F. solani*.

### 3.5. Transcriptomic Response of Fusarium solani to CEP Treatment Reveals Functional Enrichment Patterns

**Figure 5:**
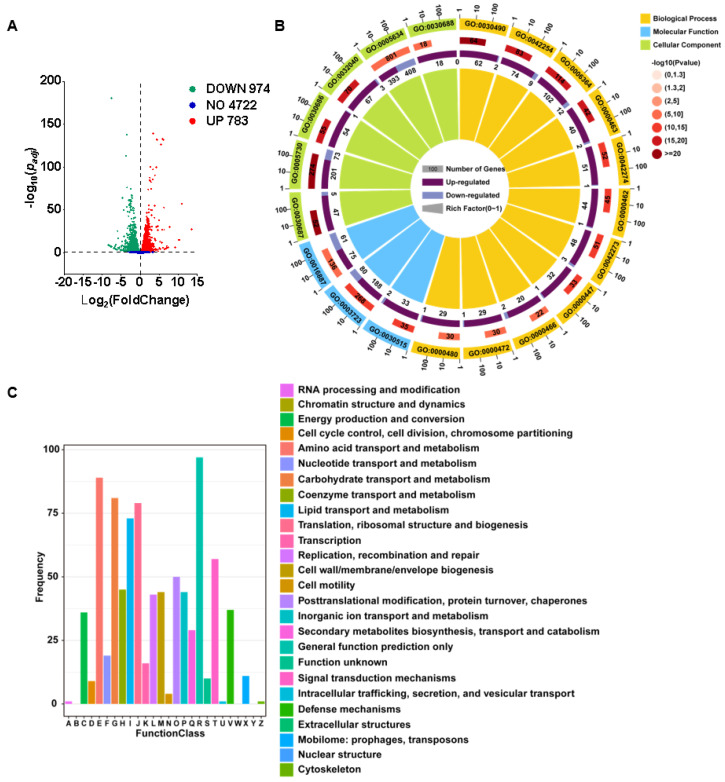
Transcriptomic analysis of *F. solani* in response to CEP treatment. (**A**) Volcano plot illustrating the distribution of differentially expressed genes in *F. solani* after CEP treatment. Red and green dots represent upregulated and downregulated genes, respectively, while blue dots indicate non-significant genes. (**B**) Gene Ontology enrichment analysis of DEGs, categorized into biological processes, molecular functions, and cellular components. The outermost ring represents the GO terms, while the inner rings show the number of differentially expressed genes, their regulation patterns, and statistical significance levels. Dark purple bars represent up-regulated genes, and light purple bars represent down-regulated genes. (**C**) Functional classification of differentially expressed genes based on the Clusters of Orthologous Groups database. Different colors represent different functional categories.

**Figure 6:**
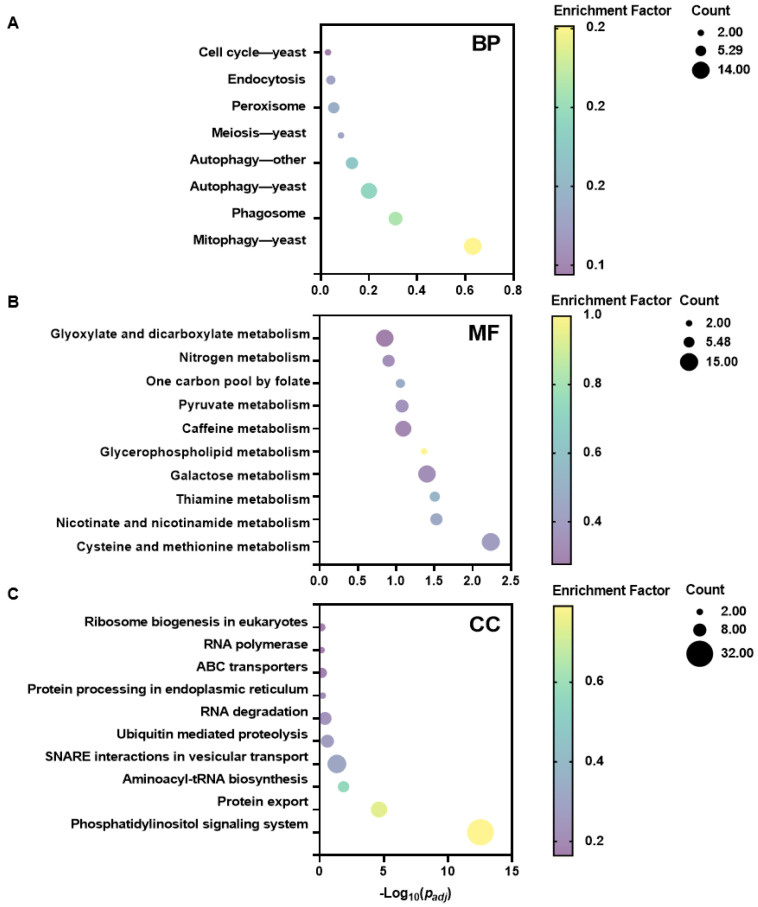
KEGG pathway enrichment analysis of differentially expressed genes in *Fusarium solani* treated with CEP. (**A**) Bubble plot showing the enrichment of differentially expressed genes in biological process (BP) categories. (**B**) Bubble plot for the enrichment of differentially expressed genes in molecular function (MF) categories. (**C**) Bubble plot presenting the enrichment of differentially expressed genes in cellular component (CC) categories. The x-axis shows the enrichment factor, and the y-axis lists pathways. The size of the bubbles indicates the count of differentially expressed genes in each pathway, and the color gradient represents the −log_10_ (*p_adj_*).

RNA-seq analysis was performed on *F. solani* treated with CEP, revealing significant changes in gene expression and specific patterns of functional enrichment among differentially expressed genes (DEGs). Compared to the control group, a total of 1757 DEGs were identified in the treated *F. solani*, including 783 upregulated and 974 downregulated genes (Figure 5A). GO (Gene Ontology) functional enrichment analysis, presented as an enrichment circle plot, categorized DEGs into cellular components (CCs), molecular functions (MFs), and biological processes (BPs), illustrating their distribution across different functional groups (Figure 5B and Table S2). DEGs were further classified based on the Cluster of Orthologous Groups (COG) database, showing enrichment across multiple COG functional categories. Notably, a higher degree of enrichment was observed in the categories of general function prediction only, amino acid transport and metabolism, carbohydrate transport and metabolism, translation, ribosomal structure and biogenesis, and lipid transport and metabolism (Figure 5C). KEGG (Kyoto Encyclopedia of Genes and Genomes) pathway enrichment analysis also covered BPs, MFs, and CCs. In the BP category, the enriched pathways included cell cycle—yeast, endocytosis, and peroxisome (Figure 6A). In the MF category, enriched pathways included glyoxylate and dicarboxylate metabolism, nitrogen metabolism, and the carbon pool by folate (Figure 6B). In the CC category, DEGs were enriched in pathways such as ribosome biogenesis in eukaryotes, RNA polymerase, and ABC transporters (Figure 6C). Overall, CEP treatment broadly affected gene expression in *F. solani*, with significant functional enrichment observed in substance transport and metabolism, genetic information processing and regulation, and various cellular life processes.

### 3.6. FsCFEM1 Is Identified as A Target of CEP Based on Molecular Docking Analysis

**Figure 7:**
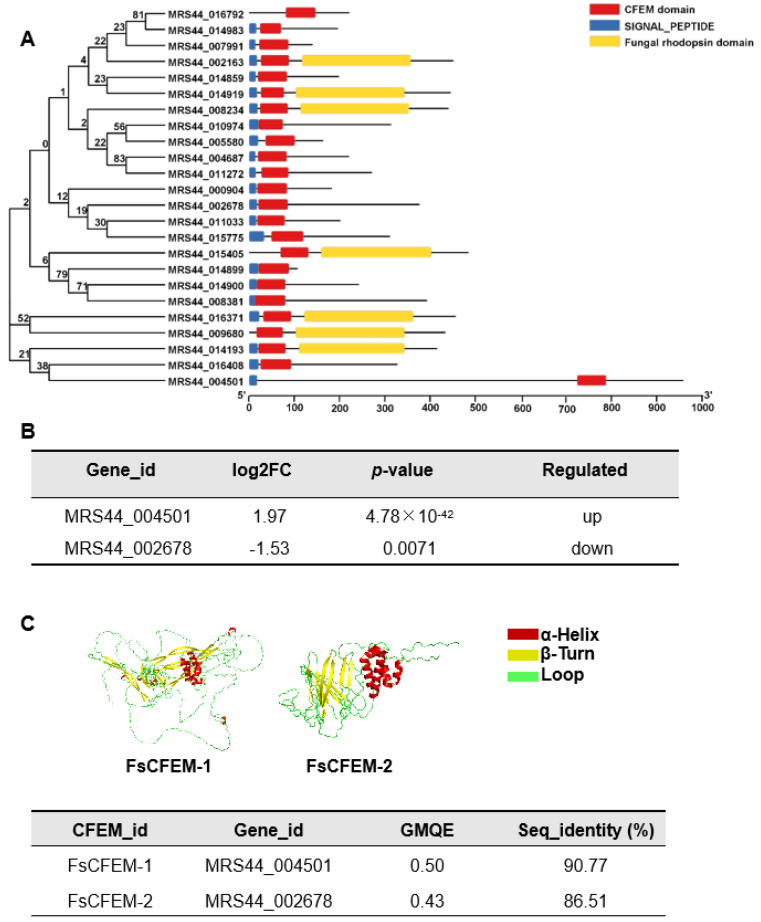
Identification and characterization of CFEM family proteins in *Fusarium solani*. (**A**) Phylogenetic tree and domain structure representation of CFEM proteins in *F. solani*. The tree shows the evolutionary relationships among CFEM proteins, and the bars represent different domains: red for the CFEM domain, blue for SIGNAL_PEPTIDE, and yellow for the fungal rhodopsin domain. (**B**) Differential expression of two CFEM genes in *F. solani* after CEP treatment. (**C**) Tertiary structure predictions of FsCFEM1 and FsCFEM2 proteins. The structures are shown with different colors, representing α-helix (red), β-turn (yellow), and loop (green). The table below provides the CFEM ID, gene ID, GMQE (Global Model Quality Estimation) value, and sequence identity (%) for each protein.

Given the critical role of CFEM proteins in fungal growth, development, and host interactions, we focused our study on the CFEM protein family. CFEM proteins play essential roles in fungal cellular physiology, including iron uptake and host immune regulation [34]. To further investigate the molecular mechanisms by which CEP affects *F. solani*, CFEM family proteins were selected for detailed analysis. All CFEM proteins in *F. solani* were identified using hmmsearch, followed by phylogenetic and domain analyses. The results showed that CFEM proteins contain a CFEM domain and a signal peptide, and some proteins also possess a fungal rhodopsin domain (Figure 7A). Transcriptomic data revealed that two genes (MRS44_004501 and MRS44_002678) exhibited significant changes in expression following CEP treatment (Figure 7B). Tertiary structure predictions were performed for the corresponding proteins, FsCFEM1 and FsCFEM2, yielding GMQE values of 0.50 and 0.43, with sequence identities of 90.77% and 86.51%, respectively. The reliability of these models supports their use in subsequent molecular docking analyses (Figure 7C).

**Figure 8:**
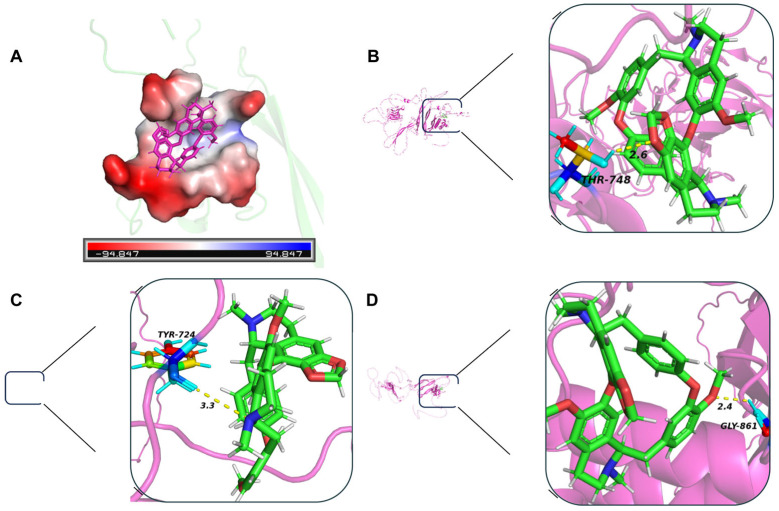
Molecular docking-derived interaction details between the FsCFEM1 protein and a small molecule in *Fusarium solani*. (**A**) Surface representation of the FsCFEM1 protein with the docked CEP small molecule (in blue). The surface is colored according to electrostatic potential (red for negative, blue for positive). (**B**–**D**) Close-up views of the binding sites within FsCFEM1. The key amino acid residues (THR748, TYR724, GLY861) involved in the interaction with the small molecule are shown, with hydrogen bonds and other interactions indicated by dashed lines and their distances (in Å) labeled.

**Figure 9:**
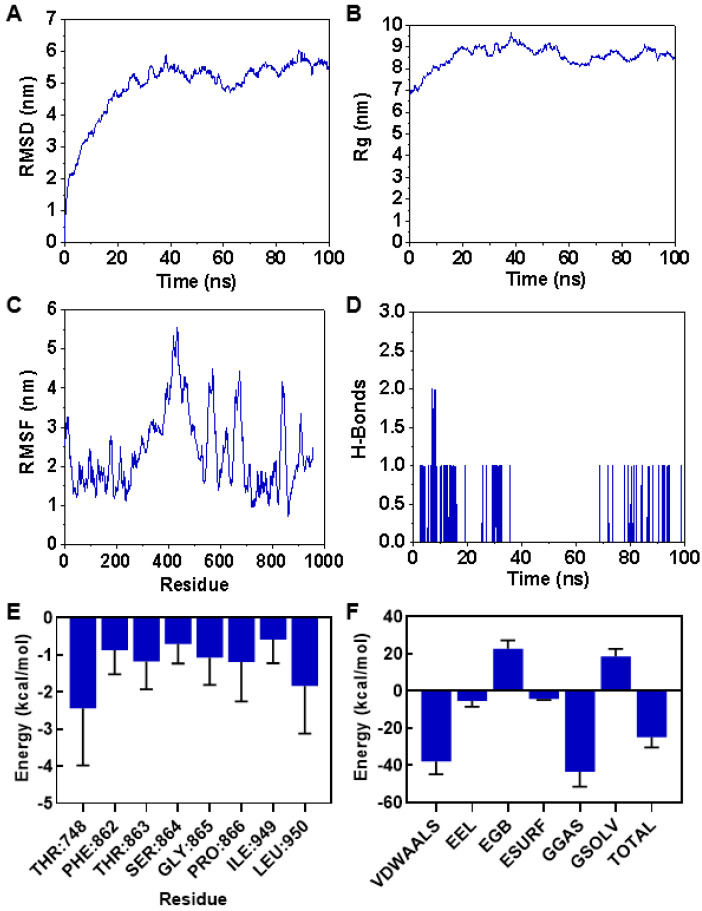
Molecular dynamics simulation analysis of the FsCFEM1-CEP complex and residue contribution analysis. (**A**) The root mean square deviation (RMSD) of the complex from its initial structure over the 100 ns simulation period. (**B**) The radius of gyration (Rg) of the FsCFEM1-CEP complex as a function of time during the 100 ns simulation. (**C**) Root mean square fluctuation (RMSF) analysis of amino acid residues. (**D**) The number of hydrogen bonds between the FsCFEM1 protein and the small molecule as a function of time during the 100 ns simulation. (**E**) Binding energy contributions of amino acid residues involved in the binding of FsCFEM1 to the small molecule. The binding energies of residues are shown in kcal/mol. (**F**) Analysis of binding free energy components. The gas-phase free energy (GGAS), total solvation free energy (GSOLV), and their components VDWAALS (van der Waals energy), electrostatic energy (EEL), polar solvation energy (EGB), non-polar solvation energy (ESURF), as well as the total binding free energy (TOTAL), are presented in kcal/mol.

Molecular docking analysis indicated an interaction between FsCFEM1 and CEP, with binding sites identified as TYR724, THR748, GLY861, etc. (Figure 8). Residue decomposition analysis showed that THR748 and LEU950 contributed significantly more to binding than other residues (Figure 9E). A binding free energy analysis revealed that the gas-phase free energy (GGAS) and both van der Waals energy (VDWAALS) and electrostatic energy (EEL) were negative, indicating that hydrophobic interactions and electrostatic forces favored binding. In the total solvation free energy (GSOLV), the non-polar solvation energy (ESURF) was negative but with a small magnitude, whereas the polar solvation energy (EGB) was positive, suggesting that the polar solvent was unfavorable for binding. The final total binding free energy was calculated as −24.86 ± 5.52 kcal/mol, confirming the interaction between the protein and the small molecule (Figure 9F). In summary, FsCFEM1 was identified as one of the potential targets of CEP in *F. solani*.

### 3.7. Molecular Dynamics Simulations Reveal the Stability and Flexibility of the CEP-FsCFEM1 Complex

Molecular dynamics (MD) simulation is a key approach for investigating the stability and dynamic properties of proteins in aqueous environments. Based on the molecular docking results, we conducted MD simulations to analyze the binding stability of the CEP-FsCFEM1 complex. During the 100 ns simulation, the radius of gyration (Rg) of the complex ranged from 6.80549 to 9.64972 nm (Figure 9B). The root mean square deviation (RMSD) exhibited slight fluctuations in the initial 40 ns but gradually stabilized over time, reaching a final RMSD value of 5.49392 nm (Figure 9A). The combined analysis of RMSD and Rg data suggests that the system tended toward stability and convergence during the simulation, indicating that CEP binding contributed to the dynamically balanced state of the complex. This structural stability may provide a foundation for the biological function of CEP. Root mean square fluctuation (RMSF) analysis was performed to assess the flexibility of amino acid residues. The results showed notable fluctuations in overall residue mobility, with particularly high fluctuations observed near residues 433, 569, 673, and 838, suggesting that CEP binding induces local conformational changes in the protein (Figure 9C). Hydrogen bond analysis between the protein and the small molecule revealed that throughout the 100 ns simulation, the value of hydrogen bonds fluctuated between 0 and 2, with an average of 0.0959 ± 0.30462 (Figure 9D). Overall, the MD simulation results indicate that the CEP-FsCFEM1 complex gradually reached a stable state over the simulation period, although certain amino acid residues exhibited a high flexibility. Furthermore, while hydrogen bonding interactions were observed, their number was relatively low and fluctuated over time. This suggests that the binding between CEP and FsCFEM1 may not be primarily driven by hydrogen bonds, and other interactions may contribute to stabilizing the complex, warranting further investigation.

## 4. Discussion

The application of machine learning provides new approaches and tools for drug screening. In recent years, machine learning algorithms have made progress in the screening of antimicrobial peptides, the prediction of antifungal activity, and drug design. Wani et al. used machine learning to build a model for predicting antimicrobial peptides. By analyzing sequence features and physicochemical properties, they screened candidate peptides with potential antimicrobial activity. Their model achieved an accuracy of 91.40% and a sensitivity of 90.05%, improving the efficiency of peptide screening [14]. Sakthivel et al. used molecular docking and virtual screening to find compounds with potential activity against cotton pests from the ZINC database, supporting the screening of natural products [15]. These studies show that machine learning is a practical method for drug screening. In this study, we built predictive models for inhibitory activity against *F. solani* using three algorithms. The Random Forest model showed the best performance, with an AUC of 0.93 (Figure 1B). The F1 score and precision of all three models were above 0.7, indicating a balanced classification between inhibitors and non-inhibitors (Figure 1F–H). This predictive method could also be applied to screen other chemical libraries for activity against different plant pathogens. Based on the model, we screened 248 candidate inhibitors from the PubChem database. Fourteen compounds had an average predicted value above 80% in all three models, including CEP, which showed a prediction accuracy of 94% under SVM (Table S1). In the follow-up research, we will conduct a comparative validation with more types of models to further assess the model’s generalizability and stability. Experimental validation confirmed that CEP inhibited *F. solani* mycelial growth by 53% at 300 mg/L and showed broad-spectrum activity (Figure 3), supporting the reliability of the machine learning model. While the results of this study demonstrate the antifungal activity of CEP in vitro, its efficacy must be validated in a host–pathogen system. This will assess its disease control and potential phytotoxicity, followed by field trials for broader evaluation. In addition, further toxicological studies, including soil microbial impact, non-target organism exposure, and environmental persistence, will be necessary to ensure the safety of CEP.

*Fusarium* spp. are widely distributed and cause significant damage as plant pathogens. In recent years, important progress has been made in the biological control of *Fusarium* spp. [35,36]. Studies have shown that several bio-based compounds exhibit strong inhibitory effects against *Fusarium* spp. For example, eugenol and carvacrol inhibit the growth of *Fusarium* spp. by disrupting its cell membrane structure and inhibiting spore germination [37,38]. Oregano essential oil (*Origanum vulgare* L.) significantly affects the pathogenicity of *F. oxysporum* f. sp. *lycopersici* [37]. Seaweed extracts inhibit *F. oxysporum’s* growth, induce plant immune responses, and can be used to control tomato wilt disease [39]. The endophytic fungi *Trichoderma asperellum* M2RT4, *Hypocrea lixii* F3ST1, and *Trichoderma harzianum* KF2R41 exhibit significant inhibitory effects against *F. oxysporum* [40]. Endophytic fungi isolated from green betel leaves (*Piper betle* L.) have been shown to significantly inhibit wilt disease caused by *F. oxysporum* [41]. Additionally, lactic acid bacteria and *Bacillus* spp. exhibit strong antagonistic effects against *F. oxysporum* [42]. This study is the first to report the inhibitory effect of CEP on *F. solani* and provides an in-depth exploration of its potential mechanisms. The experimental results show that CEP significantly inhibits the mycelial growth of *F. solani* and other plant pathogenic fungi (Figure 3A). Compared to traditional plant-derived antimicrobial substances, CEP exhibits a notable antifungal activity at low concentrations (10 mg/L), with a 53% inhibition rate observed at higher concentrations (300 mg/L) (Figure 3B). While many of the aforementioned agents act by disrupting the cell membrane, our findings suggest CEP employs a more complex mechanism. The data indicate that CEP induces oxidative stress and interferes with cell wall synthesis, potentially by targeting the FsCFEM1 protein, which represents a novel mode of action for a natural compound against *F. solani*.

CEP is a bioactive alkaloid known for its antiviral, anti-inflammatory, and anticancer properties [43]. However, its antifungal activity is not well understood. Our study found that CEP affects spore germination, oxidative metabolism, and cell wall structure in *F. solani* (Figure 4). This is similar to plant-derived compounds like thymol and carvacrol, which increase MDA levels and oxidative stress [44,45]. CEP treatment increased MDA and H_2_O_2_ content, indicating membrane damage and lipid peroxidation (Figure 4C,D). The enzyme activities of SOD, CAT, and POD were also upregulated (Figure 4F–H), suggesting that CEP induces oxidative stress, like plant essential oils. This enzymatic upregulation is likely a compensatory defense mechanism launched by the fungus to counteract the severe oxidative damage indicated by the elevated H_2_O_2_ and MDA levels. CEP also reduced chitin content in the fungal cell wall (Figure 4E), suggesting interference with cell wall synthesis. This effect is similar to known chitin synthesis inhibitors such as polyoxin and nikkomycin [46].

This study further confirms that CEP may exert its antimicrobial effect by binding to the CFEM protein in *F. solani* (Figure 8 and Figure 9). CFEM is a class of secretory proteins unique to fungi, which are closely related to iron uptake, cell adhesion, host adaptation, and pathogenicity in various plant pathogenic fungi [47,48]. Studies have shown that CFEM proteins regulate the pathogenicity of *F. oxysporum* and *B. cinerea* [49,50]. Additionally, CFEM may affect membrane protein signaling and host adaptation, with studies indicating that CFEM domains regulate the stability of G-protein-coupled receptors (GPCRs) or transmembrane proteins, thereby influencing fungal responses to environmental signals [51]. Our RNA-seq data show that, after CEP treatment, the expression of genes related to cell membrane biosynthesis, signal transduction mechanisms, and lipid transport and metabolism in *F. solani* undergo significant changes (Figure 5C). This suggests that CEP may target CFEM to affect signal transduction and membrane stability, thus inhibiting fungal growth. CFEM is also associated with fungal cell wall structure and host infection ability. Some CFEM proteins have been shown to participate in the regulation of chitin synthesis, affecting fungal cell wall stability [52]. In this study, we found that CEP treatment significantly reduced the chitin content in *F. solani* (Figure 4E), suggesting that CEP may weaken cell wall stability by targeting CFEM-related pathways, making *F. solani* more sensitive to environmental stress. Molecular docking and dynamics simulations were used to explore how CEP interacts with the FsCFEM1 protein. Docking results showed that CEP binds stably to FsCFEM1, with THR748 and LEU950 as key binding residues (Figure 9). Molecular dynamics confirmed this result. During the 100 ns simulation, the RMSD and Rg values stabilized, indicating a stable structure (Figure 9A,B). The binding energy was −24.86 ± 5.52 kcal/mol, with hydrophobic and electrostatic forces being the main contributors (Figure 9F). Hydrogen bonding played a smaller role, which is typical for alkaloid compounds. Moreover, compared to conventional antifungal targets, which are often enzymes in essential housekeeping pathways like ergosterol or chitin synthesis, targeting the FsCFEM1 protein represents a new strategy. Because CFEM proteins often function as effectors critical for interaction with the host, inhibiting CFEM proteins may offer a more precise approach to disrupting a fungus’s virulence. This proposed mechanism is preliminary, as it relies on transcriptomic and computational predictions that both await direct experimental validation.

## 5. Conclusions

In this study, we used machine learning and virtual screening to identify CEP as a potential antifungal compound against *F. solani*. Experiments showed that CEP effectively inhibited mycelial growth, spore production, and germination and disrupted the fungal redox balance. Transcriptome analysis showed changes in gene expression related to basic metabolic pathways, suggesting an effect on fungal physiology. Molecular docking and dynamics simulations indicated that CEP binds stably to FsCFEM1, mainly through residues THR748 and LEU950. These results highlight the novelty of CEP as a candidate and a new mechanism targeting the FsCFEM1 protein. However, acknowledging the limitations of relying on in vitro and computational data, the next steps must include validation in vivo and the development of a formulation to assess its translational potential in plant disease control. Future strategies could also focus on enhancing CEP’s bioavailability through nanoformulations or encapsulation.

## Figures and Tables

**Table 1:** Prediction results of the three machine learning models in the training set.

Model	Accuracy	Precision	Recall	F1 Score
RF	0.88	0.86	0.85	0.83
SVM	0.79	0.80	0.75	0.78
NN	0.82	0.80	0.78	0.78

## Data Availability

The original contributions presented in this study are included in the article/Supplementary Materials. Further inquiries can be directed to the corresponding author.

## Supplementary Materials

The following supporting information can be downloaded at: https://www.mdpi.com/article/10.3390/microorganisms13061423/s1, Figure S1: The variation of model performance metrics with the number of features in RFE analysis; Figure S2: Correlation matrix plot of the 50 features selected by RFE; Figure S3: Microscopic morphology of Fusarium solani hyphae and spores; Table S1: Predicted compound in this study; Table S2: GO numbers and their descriptions shown in the enrichment circle diagram.
