# Supplementary material for: Cepharanthine Inhibits Fusarium solani via Oxidative Stress and CFEM Domain-Containing Protein Targeting
Source: Microorganisms. 2025 Jun 18;13(6):1423. doi: 10.3390/microorganisms13061423 (PMC12196386; doi:10.3390/microorganisms13061423)
Supplement: Supplementary file 1 [file microorganisms-13-01423-s001.zip › microorganisms-3671064-supplementary.pdf]

# Supplementary Data

**Table S1.** Predicted compound in this study.

| PubChem CID | IUPAC Name | CAS | RF | SVM | NN |
|---|---|---|---|---|---|
| 73078 | Tetrandrine | 518-34-3 | 0.86 | 0.96 | 1.00 |
| 10206 | Cepharanthine | 481-49-2 | 0.80 | 0.94 | 1.00 |
| 159795 | Thalidasine | 16623-56-6 | 0.84 | 0.97 | 1.00 |
| 100231 | Obaberine | 1263-80-5 | 0.87 | 0.97 | 1.00 |
| 100230 | Thalicberine | 5096-71-9 | 0.87 | 0.96 | 1.00 |
| 442333 | Oxyacanthine | 548-40-3 | 0.81 | 0.90 | 1.00 |
| 175902 | Gyrocarpine | 102487-16-1 | 0.82 | 0.90 | 1.00 |
| 442366 | Thalmine | 7682-65-7 | 0.83 | 0.91 | 1.00 |
| 197726 | Isochondrodendrine | 477-62-3 | 0.86 | 0.89 | 1.00 |
| 72343 | Hernandezine | 6681-13-6 | 0.81 | 0.98 | 1.00 |
| 159911 | Guattegaumerine | 21446-35-5 | 0.89 | 0.91 | 1.00 |
| 440585 | Berbamunine | 485-18-7 | 0.89 | 0.91 | 1.00 |
| 73053 | Phaeanthrine | 27670-80-0 | 0.90 | 1.00 | 1.00 |
| 73400 | Dauricine | 524-17-4 | 0.92 | 0.98 | 1.00 |

**Table S2.** GO numbers and their descriptions shown in the enrichment circle diagram.

| ID | Description |
|---|---|
| GO:0030687 | preribosome |
| GO:0005730 | nucleolus |
| GO:0030490 | maturation of SSU-rRNA |
| GO:0042254 | ribosome biogenesis |
| GO:0006364 | rRNA processing |
| GO:0000463 | maturation of LSU-rRNA from tricistronic rRNA |
| GO:0030686 | 90S preribosome |
| GO:0032040 | small-subunit processome |
| GO:0042274 | ribosomal small subunit biogenesis |
| GO:0000462 | maturation of SSU-rRNA from tricistronic rRNA |
| GO:0042273 | ribosomal large subunit biogenesis |
| GO:0030515 | snoRNA binding |
| GO:0003723 | RNA binding |
| GO:0000447 | endonucleolytic cleavage |
| GO:0000466 | maturation of 5.8S rRNA from tricistronic rRNA |
| GO:0005634 | nucleus |
| GO:0000472 | endonucleolytic cleavage to generate mature 5'-end of SSU-rRNA |
| GO:0000480 | endonucleolytic cleavage in 5'-ETS of tricistronic rRNA |
| GO:0016887 | ATP hydrolysis activity |
| GO:0030688 | preribosome, small subunit precursor |

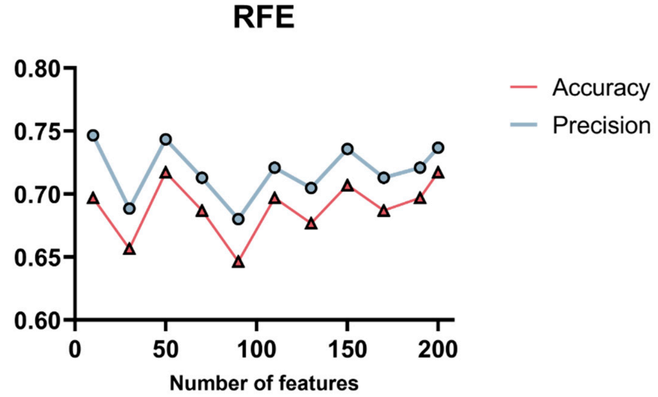


**Figure S1. The variation of model performance metrics with the number of features in RFE analysis.** During the RFE iteration process, the model performance was better when the number of features was 50.

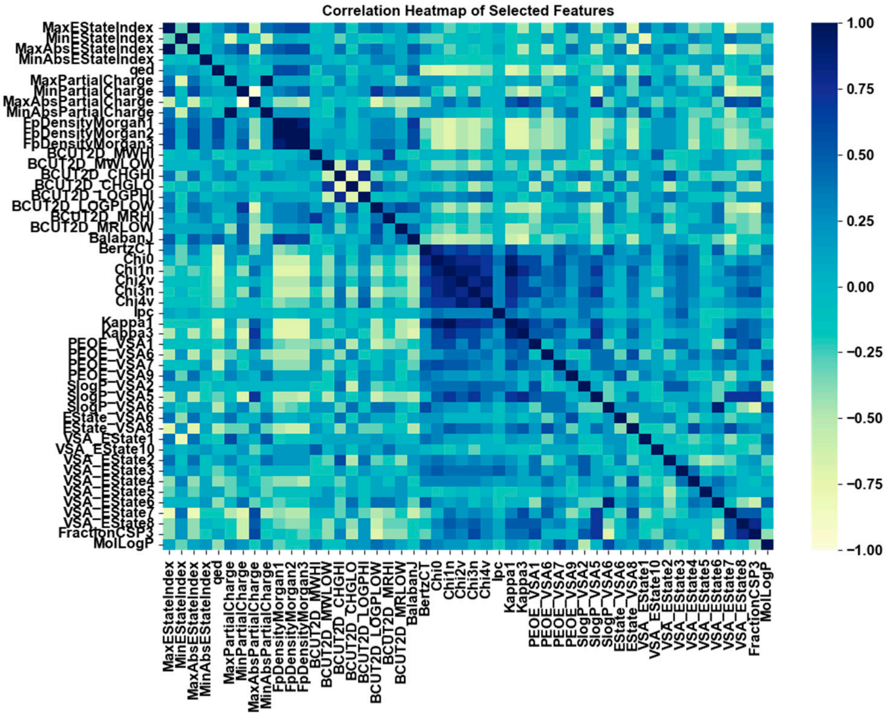


**Figure S2. Correlation matrix plot of the 50 features selected by RFE.** The Pearson correlation coefficient values are mostly in the range of -0.5 to 0.5, indicating that the linear correlation between most feature pairs is weak and features retained by RFE effectively removed redundancy.

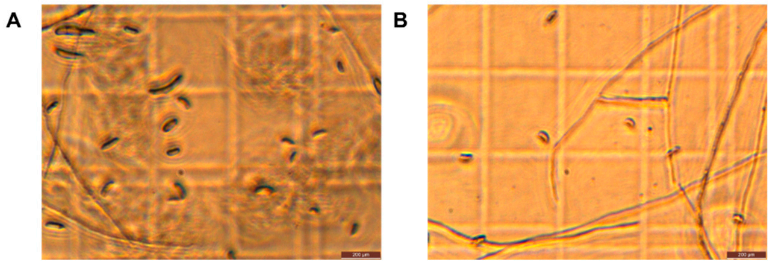


**Figure S3. Microscopic morphology of *Fusarium solani* hyphae and spores.** (A) Untreated control. (B) Culture treated with 200 mg/L CEP. Bars=200μm.
